# Lung Cancer and Elemental Carbon Exposure in Trucking Industry Workers

**DOI:** 10.1289/ehp.1204989

**Published:** 2012-06-01

**Authors:** Eric Garshick, Francine Laden, Jaime E. Hart, Mary E. Davis, Ellen A. Eisen, Thomas J. Smith

**Affiliations:** 1 Pulmonary and Critical Care Medicine Section, VA Boston Healthcare System, Boston, Massachusetts, USA; 2 Channing Division of Network Medicine, Brigham and Women’s Hospital, Harvard Medical School, Boston, Massachusetts, USA; 3 Exposure, Epidemiology and Risk Program, Department of Environmental Health, Harvard School of Public Health, Boston, Massachusetts, USA; 4 Department of Epidemiology, Harvard School of Public Health, Boston, Massachusetts, USA; 5 Department of Urban and Environmental Policy and Planning, Tufts University, Medford, Massachusetts, USA; 6 Environmental and Occupational Medicine and Epidemiology Program, Department of Environmental Health, Harvard School of Public Health, Boston, Massachusetts, USA; 7 Environmental Health Sciences Division, School of Public Health, University of California, Berkeley, California, USA

**Keywords:** diesel exhaust, elemental carbon, lung cancer, occupational exposure, particulate matter, traffic

## Abstract

Background: Diesel exhaust has been considered to be a probable lung carcinogen based on studies of occupationally exposed workers. Efforts to define lung cancer risk in these studies have been limited in part by lack of quantitative exposure estimates.

Objective: We conducted a retrospective cohort study to assess lung cancer mortality risk among U.S. trucking industry workers. Elemental carbon (EC) was used as a surrogate of exposure to engine exhaust from diesel vehicles, traffic, and loading dock operations.

Methods: Work records were available for 31,135 male workers employed in the unionized U.S. trucking industry in 1985. A statistical model based on a national exposure assessment was used to estimate historical work-related exposures to EC. Lung cancer mortality was ascertained through the year 2000, and associations with cumulative and average EC were estimated using proportional hazards models.

Results: Duration of employment was inversely associated with lung cancer risk consistent with a healthy worker survivor effect and a cohort composed of prevalent hires. After adjusting for employment duration, we noted a suggestion of a linear exposure–response relationship. For each 1,000-µg/m^3^ months of cumulative EC, based on a 5-year exposure lag, the hazard ratio (HR) was 1.07 [95% confidence interval (CI): 0.99, 1.15] with a similar association for a 10-year exposure lag [HR = 1.09 (95% CI: 0.99, 1.20)]. Average exposure was not associated with relative risk.

Conclusions: Lung cancer mortality in trucking industry workers increased in association with cumulative exposure to EC after adjusting for negative confounding by employment duration.

Concern has been raised for > 40 years that exposure to diesel exhaust results in lung cancer in humans (National Research Council 1981). Diesel exhaust has included a mixture of elemental carbon (EC) particles with adsorbed mutagenic and carcinogenic polycyclic aromatic hydrocarbons (PAH), condensed engine oil, other organic compounds, and exhaust gases. Previous epidemiologic studies have reported a positive association between lung cancer risk and work in occupations with some degree of diesel exhaust exposure [e.g., Bhatia et al. 1998; Diesel Working Group 1995; Garshick et al. 2004; Lipsett and Campleman 1999; Steenland et al. 1990; U.S. Environmental Protection Agency (EPA) 2002].

Previous evaluations by administrative and regulatory authorities have judged diesel exhaust to be a probable lung carcinogen (International Agency for Research on Cancer 1989) likely capable of causing lung cancer at ambient levels [California Environmental Protection Agency 1998; U.S. EPA 2002]. The main criticisms have been that few studies have included exposure measurements, and those studies that have included such measures have been limited in their ability to credibly extrapolate changes in exposure over time, to link exposure to personal work records, or to provide quantitative risk estimates (Health Effects Institute Air Toxics Review Panel 2007). In the United States increasingly stringent emission standards have been implemented for new on-road and off-road diesel engines (U.S. EPA 2001, 2007). However, older diesel engines have remained in service for several decades in the United States and for even longer in other parts of the world. Therefore, understanding the quantitative assessment of lung cancer risk remains important.

Accurate linkage between work history and quantitative or semiquantitative estimates or exposure categories requires knowledge of current and historical exposure scenarios and estimates of intensity. To address this issue, we designed a retrospective cohort study and a current and historical exposure assessment of workers employed by four large unionized (Teamsters) carriers in the United States. Our approach takes advantage of three key characteristics: *a*) the large number of detailed work records available, *b*) stable relationships between union job titles and duties over time, and *c*) known dates of diesel equipment use (Garshick et al. 2002). We conducted a national exposure assessment in this industry between 2001 and 2006 to characterize exposures to EC as a marker of exposure to diesel and other traffic-related particulate matter (PM) (Davis et al. 2006, 2007, 2009; Smith et al. 2006) including a source apportionment assessment for components of exposure (Sheesley et al. 2008a, 2008b). EC was chosen as our exposure marker because it is an exhaust constituent that may serve as a tracer for diesel emissions when the source is close to the subject, as it is in trucking operations. Exposure models were used to estimate job- and location-specific exposures to EC in the United States between 1971 and 2000, taking into account spatial and temporal determinants (Davis et al. 2006).

We have previously reported elevated standardized mortality ratios (Laden et al. 2007) and an elevated risk of lung cancer mortality with increasing years of work as a driver or dockworker in this cohort (Garshick et al. 2008). For the present analysis we linked a historical EC exposure model to work records to estimate personal EC exposures and examine their association with lung cancer mortality.

## Methods

*Population and work history.* Computerized work records were obtained for 58,326 unionized trucking industry employees (54,319 men and 4,007 women) employed in 1985 in four large national trucking companies. Job titles and duties were common among all companies, and records included dates of hire and change in employment status, job, and trucking terminal assignment. In one company (18.5% of the cohort) complete records were only available for workers still employed in 1993, so that company’s workers were only included if they were continuously employed from 1985 to 1993. Records for another company were not available before 1972, and work history before that date was based on job and terminal in 1972. Approval was obtained from the institutional review boards of Brigham and Women’s Hospital, Harvard School of Public Health, and VA Boston Healthcare System. Individual consent to participate was not required.

*Mortality.* Date of death and cause-specific mortality from 1985 through 2000 was obtained from the National Death Index using social security number, name, and date of birth as matching criteria. Because prolonged survival is rare, we included deaths with primary lung cancer, according to the *International Classification of Diseases, 9th Revision* (ICD-9) code 162 (World Health Organization 1978) and *10th Revision* (ICD-10) codes C33-C34 (World Health Organization 1993) as cases even if not the underlying cause of death. Men were included in the analysis if they worked for at least 1 year for one of the study companies. In addition, analyses were limited to men ≥ 40 years of age in 1985 because most lung cancer deaths (96%) occurred in this age group (*n* = 31,135).

*Current exposure.* Major jobs, diesel use, and numbers of workers in each job in 1985 are presented in Table 1. An exposure assessment was conducted from 2001 through 2006. This assessment included 5-day sampling trips to 36 trucking terminals randomly chosen to be representative of the 139 large terminals (> 100 workers) in these companies (Davis et al. 2006; Smith et al. 2006). We measured cross-shift (8–12 hr) EC in particulate matter ≤ 1.0 μm in aerodynamic diameter (PM_1.0_) the using the 5040 method from the National Institute for Occupational Safety and Health (1998). We collected > 4,000 EC measurements, including personal samples from dock workers and mechanics, and area measurements in loading docks, offices, and truck cabs. Ambient background conditions were measured at the periphery of the upwind terminal. Area measurements, including 214 loading dock samples, were collected at 44 smaller terminals (1–2 per trip) within 75 miles.

**Table 1:** Distribution of jobs in 1985 among men ≥ 40 years of age with ≥ 1 year of work (*n* = 31,135).

Job in 1985	Job duties	n (%)
Long-haul driver (LH)		Drives heavy-duty trucks between large terminals. Diesel first used 1951–1957; transition complete 1954–1965, depending on company		10,825 (35)
Pick-up/delivery (P&D)		Drives trucks locally; picks up and delivers cargo. Diesel first used 1972–1978; transition complete 1980–1992, depending on company		5,866 (19)
Dockworker		Loads/unloads cargo on dock using forklifts		5,710 (18)
		Diesel forklifts in three companies starting in 1979–1986 through 1994–1996 in large terminals		
Combination worker		Duties include P&D driver and dockworker		4,938 (16)
Mechanic		Maintains trucks in large terminal shops		1,741 (6)
Hostler		Drives a tractor or a specialized tractor to move trailers to and from freight dock and in yard		666 (2)
Clerk		Cashiers, dock clerks, dispatchers, customer service representatives, and others in terminal offices		843 (3)
Other jobs		Janitor, unionized manger, trainee, or not defined		546 (2)

Separate exposure models were constructed for drivers and terminal workers. Drivers included pick-up and delivery (P&D) drivers, long-haul (LH) drivers, and hostlers (Davis et al. 2007) and terminal-based workers included dockworkers and mechanics (Davis et al. 2006). Models were validated with six repeat trips (Davis et al. 2009). Using structural equation modeling (SEM) for the terminal workers, personal EC exposure was a function of work area EC, and in turn, work area EC was a function of job, terminal characteristics related to size and ventilation, and upwind ambient (background) EC. Background EC was a function of local weather, distance from an interstate highway, land use, and U.S. census region. For P&D and LH drivers, home terminal ambient EC was a significant determinant of truck cab EC. In contrast to LH drivers who had air conditioned cabs, temperature was also a determinant of exposure for P&D drivers because cab EC was higher when the windows were predicted to be open. For drivers and hostlers, we determined factors scaling exposure based on ambient terminal EC. Office workers were assumed to have background exposures.

*Historical exposure.* Details of our historical model are presented elsewhere (Davis et al. 2011). Historical trends in ambient terminal EC were modeled based on historical trends in the coefficient of haze (COH) available for 1971 through 2000, a measurement of particulate matter based on optical density, highly predictive of ambient EC (*R*^2^ = 0.94) (Cass et al. 1984). We validated this approach using data from a 1988 through 1989 assessment of EC in the same industry that used the same methodology as our current assessment (Zaebst et al. 1991). The ratio of ambient EC from 1988–1989 compared with 2001–2006 was identical to the ratio of COH data over the same time periods; this finding demonstrates that historical trends in COH and ambient EC were similar (Davis et al. 2011). Monthly ambient background EC estimates were determined for the 139 large terminals between 1971–2000 using location specific temperature, windspeed, and other terminal-specific data as input into the SEM exposure model.

In addition to changes in ambient exposure, our historical model accounted for changes in job-related exposures based on comparison of 1988 through 1989 to 2001 through 2006 measured values. For dockworkers who drove propane forklifts, the geometric mean of predicted EC in terminals that were sampled in 1988–1989 and adjusted for background EC was 1.36 µg/m^3^ (Davis et al. 2011; Zaebst et al. 1991). The value measured by Zaebst et al. (1991) was nearly identical (1.30 µg/m^3^), supporting the validity of our approach. The data reported by Zaebst et al. (1991) were also used to account for changes in the type of fuel used for forklifts, which included diesel in large terminals operated by three companies during the 1980s through the mid-1990s, as well as gasoline and propane (Table 1). This approach provided multipliers that reflect historical changes in exposure for each job, including fuel type for dockworkers. We used these multipliers to estimate exposures for 1971 through 1989; we applied linear extrapolation for exposures that occurred from 1990 through 2000.

*Personal exposure.* Data on historical job and terminal-specific monthly EC concentrations, which were determined by the exposure model, were summed by year to estimate an individual’s cumulative exposure (µg/m^3^-months). Because workers may have had more than one job in a year, job-specific EC was weighted by the fraction of time spent in each job. Job-specific EC values before 1971 (8% of total exposure time) were assigned 1971 exposures because COH data were not available to estimate background. Combination workers (Table 1) were assumed to spend 50% of their time as a P&D driver and 50% as a dockworker. Fifty percent of exposure time was accounted for by work at the 139 larger terminals for which we had directly modeled exposure. EC predictions at one terminal were an order of magnitude higher than in others due to extreme weather values and other model input characteristics. Therefore, this terminal was assigned exposure values from another terminal in the same city. Based on available information, it is likely that nearly all terminals not directly modeled were smaller terminals (≤ 100 workers). From our exposure assessment, the mean difference between large and smaller terminal loading dock area EC measured during the same trip was 0, indicating no meaningful bias in applying our terminal exposure model. For exposure time without directly modeled estimates, exposure was assigned in one of three ways: for terminals still in existence, exposure was assigned using the nearest directly modeled larger terminal (25% of exposure time; 1,041 terminals); for terminals no longer in operation but with known region, exposure was assigned using the average exposure of directly modeled terminals in the same region (24% of exposure time; 1,583 terminals), or national annual job averages if the location of the terminal was not known (1% of exposure time; 83 terminals). Although historical data were limited, it was assumed that gasoline forklifts were used before 1971. Exposures for dockworkers and combination workers for 1971 and afterward were assigned based on company-specific information on the use of diesel or propane forklift fuel. Workers were not assigned any EC exposure after retirement or before hire.

*Statistical methods.* Proportional hazard regression was used to estimate associations between lung cancer mortality and EC. To closely adjust for age and lung cancer secular trends, risk sets were generated using attained age in 1-year increments as the timeline, and an ordinal variable for calendar year (1985 through 2000) was included in all models. Workers in the cohort were hired over many decades (from before 1960 until after 1980). Each period of work was associated with different unmeasured work practices and vehicle characteristics. Furthermore, individuals who entered follow-up (in 1985) at different ages had different risks of developing lung cancer during the follow-up period. To meet the assumptions of proportional hazards, we assigned separate baseline hazards based on decade of hire (< 1960, 1960–1969, 1970–1979, ≥ 1980) and age in 1985 (40 to < 50, 50 to < 60, 60 to < 70, ≥ 70 years). For example, the baseline hazard for a person 40 years of age in 1985 (born in 1945) who began work in 1975 was the same as that for all workers in their 40s in 1985 who were also hired in the 1970s, and the risk set for that individual would include all workers with the same attained age. All models were also adjusted for race and census region of residence (based on last known home address). We conducted sensitivity analyses with and without total years of employment as a time-dependent covariate (modeled as either continuous or in quartiles) to assess its effect as a potential confounder. Previously in this cohort, Garshick et al. (2008) reported that lung cancer mortality was inversely associated with total duration of employment; this association is consistent with a survivor bias among those who remained at work.

Cumulative EC (µg/m^3^-months) and average EC (micrograms per cubic meter) were estimated for each worker from date of hire through the earlier of retirement date or through 31 December 2000. Time-dependent cumulative exposure was modeled as lags of 0, 5, and 10 years, as continuous, and in quartiles. Time-dependent average exposure was modeled with lags of 0 and 5 years (continuous and in quartiles). For example, in a 5-year lag model, exposure in the current year and in the previous 4 years is not included in exposure calculations to exclude the contribution of more recent exposure to lung cancer mortality risk. Linear trend *p*-values were derived using an ordinal variable that was based on the median of each quartile. Penalized splines were incorporated into regression models to assess possible nonlinearity in mortality risk. Analyses were performed using SAS software (version 9.2; SAS Institute Inc., Cary, NC) or R for Unix (R Foundation for Statistical Computing, Vienna, Austria).

Analyses were also performed that excluded workers who were employed for ≥ 1 year as a mechanic (*n* = 1,811). Mechanics experienced significant historical changes in job duties that weaken the validity of extrapolation of current exposure to historical estimates. Our exposure estimates reflect the current practice of performing minor repairs and maintenance whereas in the past major engine repairs were performed. In addition, the nature of exposure is different from that of other workers. Based on our field observations, mechanics are exposed to EC for relatively brief periods when engines are intermittently run inside shops allowing for particle aging. Workers in other jobs on roadways and loading docks have exposures characterized by considerably longer periods of exposure to fresh exhaust from continuously running engines.

## Results

*Population.* The mean cumulative years of work in the study cohort was 21.6 years, 84.9% were Caucasian, and most lived in the South (37.5%) or Midwest (31.5%). (Table 2). Long-haul diesel trucks were first used during the 1950s in this industry, and nearly all workers began employment during or after this period. Most workers also began employment concurrent with or after the introduction of diesel P&D trucks first used during the 1970s. There were 4,003 dockworkers or combination workers with at least 1 year of diesel forklift exposure (median, 5.8 years; interquartile range, 2.9–9.0 years) in the 1980s and into the 1990s. The average age of hire was in the mid-30s (Table 2), most likely due to hiring policies requiring previous experience. There was a wide range of cumulative EC exposure (Table 3). The 75th percentile of estimated cumulative exposure was > 8 times that of the 25th percentile for a 10-year lag, and almost 4 or 5 times higher for no lag and 5-year lagged exposures, respectively.

**Table 2:** Descriptive characteristics of the 31,135 male trucking industry workers [mean ± SD or *n* (%)].

Characteristic	Value
Age at hire (years)	36.0 ± 8.3
Age in 1985 (years)	49.1 ± 6.0
Years of work	21.6 ± 8.7
< 10	2,950 (9.5)
10 to < 20	10,443 (33.5)
20 to < 30	12,202 (39.2)
≥ 30	5,540 (17.8)
Race	
Caucasian	26,430 (84.9)
African American	2,818 (9.1)
Other/unknown	1,887 (6.1)
Census regiona	
Northeast	4,555 (14.6)
South	11,682 (37.5)
Midwest	9,818 (31.5)
West	4,759 (15.3)
Unknown	321 (1.0)
Decade of hire	
< 1960	3,107 (10.0)
1960–1969	8,284 (26.6)
1970–1979	13,745 (44.2)
≥ 1980	5,999 (19.3)
aBased on last known home address.	

**Table 3:** Distribution of cumulative EC (µg/m^3^-months) from hire date through retirement or through 31 December 2000, whichever occurred first, in the entire cohort, excluding 1,811 mechanics.

Entire cohort (n = 31,135)					Cohort excluding mechanics (n = 29,324)				
Percentile	No lag	5-year lag	10-year lag	No lag	5-year lag	10-year lag
Maximum		24,130		24,130		23,106		15,242		15,074		10,341
99		8,725		7,729		6,482		6,623		5,588		4,543
95		4,829		4,305		3,579		4,044		3,599		2,997
90		3,560		3,131		2,595		3,057		2,723		2,283
75		2,076		1,803		1,436		1,891		1,635		1,305
50		1,061		860		596		990		801		553
25		530		371		167		508		351		155
10		271		136		2.0		260		128		0
5		167		48		0		161		44		0
Minimum		10		0		0		10		0		0

*Lung cancer mortality.* Through 2000, there were 779 lung cancer cases (734 underlying cause) out of 4,306 deaths. In models adjusted for calendar year, race, and census region, we examined cumulative EC in quartiles without and with adjustment for employment duration (Table 4). When adjusted for race, calendar year, and census region only, lung cancer mortality was elevated for the upper three cumulative EC quartiles compared with the lowest quartile, but differences were not statistically significant [hazard ratio (HR) = 1.17–1.19 for 5-year lagged exposures, excluding mechanics]. Associations with cumulative EC exposure were stronger for all three lags after adjusting for duration of employment but did not display a monotonic dose response when estimated for the cohort as a whole. However, HRs for 5- and 10-year lagged exposures increased with each quartile when mechanics were excluded, which resulted in estimated HR of 1.48 [95% confidence interval (CI): 1.05, 2.10] and 1.41 (95% CI: 0.95, 2.11) for the highest versus lowest quartiles of 5- and 10-year lagged exposures, respectively. Associations were weaker for average EC exposure (5-year lag shown as an example) in models adjusting for duration of employment (Table 4).

**Table 4:** Lung cancer HRs associated with each quartile of cumulative and average EC, with and without adjustment for duration of work.

Entire cohort (n = 31,135)							Cohort excluding mechanics (n = 29,324)						
Exposure	Person-years	Lung cancer deaths	HR (95% CI) unadjusted for duration of work	HR (95% CI) adjusted for duration of work	Person-years	Lung cancer deaths	HR (95% CI) unadjusted for duration of work	HR (95% CI) adjusted for duration of work
Cumulative EC (µg/m3-months)															
No lag															
< 530	106,272		153		Reference		Reference		105,868		153		Reference		Reference
530 to < 1,061	106,208		194		1.13 (0.90, 1.42)		1.24 (0.98, 1.57)		105,312		193		1.13 (0.90, 1.42)		1.25 (0.99, 1.60)
1,061 to < 2,076	106,149		209		1.14 (0.89, 1.47)		1.30 (0.99, 1.70)		102,534		202		1.13 (0.87, 1.46)		1.30 (0.99, 1.72)
≥ 2076	106,039		223		0.98 (0.74, 1.29)		1.16 (0.86, 1.57)		86,600		193		1.02 (0.76, 1.36)		1.24 (0.89, 1.71)
p for trend					0.37		0.92						0.63		0.71
5-year lag															
< 371	106,359		122		Reference		Reference		105,513		122		Reference		Reference
371 to < 860	106,223		193		1.18 (0.92, 1.51)		1.30 (1.01, 1.68)		104,909		191		1.18 (0.92, 1.52)		1.31 (1.01, 1.71)
860 to < 1,803	106,114		208		1.16 (0.88, 1.53)		1.35 (1.01, 1.81)		102,496		202		1.17 (0.88, 1.55)		1.38 (1.02, 1.87)
≥ 1,803	105,972		256		1.12 (0.83, 1.52)		1.36 (0.98, 1.89)		87,397		226		1.19 (0.86, 1.63)		1.48 (1.05, 2.10)
p for trend					0.97		0.39						0.61		0.16
10-year lag															
< 167	106,409		114		Reference		Reference		104,329		112		Reference		Reference
167 to < 596	106,213		183		1.04 (0.79, 1.37)		1.14 (0.86, 1.52)		104,487		179		1.06 (0.80, 1.40)		1.17 (0.88, 1.57)
596 to < 1,436	106,120		205		1.01 (0.74, 1.37)		1.18 (0.85, 1.64)		102,712		202		1.05 (0.77, 1.45)		1.26 (0.90, 1.78)
≥ 1,436	105,926		277		1.03 (0.72, 1.45)		1.25 (0.86, 1.82)		88,786		248		1.12 (0.78, 1.61)		1.41 (0.95, 2.11)
p for trend					0.96		0.39						0.57		0.15
Average exposure, 5-year lag (µg/m3)															
< 3.6	106,304		146		Reference		Reference		105,930		146		Reference		Reference
3.6 to < 5.4	106,153		211		1.15 (0.93, 1.43)		1.15 (0.93, 1.43)		105,950		211		1.15 (0.93, 1.43)		1.15 (0.93, 1.43)
5.4 to < 7.9	106,093		221		1.11 (0.89, 1.39)		1.12 (0.90, 1.40)		105,512		221		1.11 (0.89, 1.39)		1.12 (0.89, 1.40)
≥ 7.9	106,117		201		1.06 (0.84, 1.34)		1.08 (0.85, 1.36)		82,922		163		1.11 (0.87, 1.43)		1.13 (0.88, 1.44)
p for trend					0.97		0.88						0.61		0.53
All models adjusted for race, calendar year of follow-up, and census region.															

Excluding the mechanics, regression models incorporating penalized splines for cumulative EC indicated that lung cancer risk increased with increasing cumulative EC and did not statistically significantly depart from linearity. For each 1,000 µg/m^3^-months of cumulative EC, the estimated HR is 1.04 (95% CI: 0.97, 1.11), 1.07 (95% CI: 0.99, 1.15), and 1.09 (95% CI: 0.99, 1.20) for no exposure lag, a 5-year exposure lag, and a 10-year exposure lag, respectively (Table 5). There was no association with average EC as a continuous covariate (*p* = 0.71 for the no lag and *p* = 0.69 for the 5-year lag model) (data not shown).

**Table 5:** Lung cancer hazard per 1,000 µg/m^3^-months associated with cumulative EC for exposure with no lag, a 5-year lag, and a 10-year lag, excluding mechanics.

Coefficient (SE) 1,000 µg/m3-months	Relative hazard	95% CI
No lag	0.0345 (0.0349)	1.04	0.97, 1.11
5-year lag	0.0665 (0.0379)	1.07	0.99, 1.15
10-year lag	0.0849 (0.0501)	1.09	0.99, 1.20
Adjusted for race, census region, calendar year of follow-up, and duration of employment. For the no lag, 5-year lag, and 10-year log, p = 0.32, 0.08, and 0.09, respectively.			

Duration of employment was correlated with cumulative EC (*r* = 0.55–0.74, depending on lag), but lung cancer mortality risk statistically significantly decreased with duration. For example, in the 5-year lag cumulative EC model excluding mechanics, the HR per year of work was 0.97 (95% CI = 0.96, 0.99). The attenuation of relative risk estimates if employment duration was not included is consistent with negative confounding.

## Discussion

We performed a retrospective assessment of EC exposure and lung cancer mortality in a large U.S. trucking industry cohort and found that estimated cumulative exposure to EC was positively associated with lung cancer mortality after adjusting for employment duration in addition to race, census region, and calendar year. Other than the mechanics, workers with occupational EC exposure were in driver- or loading dock–related jobs that involved regular and often continuous exposure to vehicle exhaust from diesel and nondiesel sources. Estimated relative risks were stronger when 1,811 mechanics were excluded. There was a suggestion of a linear exposure–response relationship, and the strongest associations were observed with a 5- and 10-year cumulative exposure lag with a HR of 1.07 (95% CI: 0.99, 1.15) and 1.09 (95% CI: 0.99, 1.20), respectively. Average exposure was not associated with lung cancer mortality.

Estimated relative risks decreased with employment duration, which suggests that duration was a surrogate for time-varying health status. This finding was likely due to bias caused by left truncation in a cohort composed of prevalent hires combined with a healthy worker survivor effect. Left truncation occurs when subjects who were hired before the start of follow-up are included. Inclusion of such prevalent hires and variability in susceptibility to exposure has been shown to cause downward bias between disease and exposure duration (Applebaum et al. 2011). Effects of left truncation were observed in a study of lung cancer and silica exposure where exposure was inversely associated with lung cancer mortality among prevalent hires, despite greater cumulative exposure (Applebaum et al. 2007). We also observed an inverse association between employment duration and lung cancer mortality in a cohort of prevalent hires in the U.S. railroad industry (Garshick et al. 2004).

The healthy worker survivor effect is commonly presumed to be small or absent for lung cancer. However, results from occupational lung cancer studies of arsenic, radiation, and other diesel-exposed workers suggest otherwise (Arrighi and Hertz-Picciotto 1996; Brown et al. 2004; Cardis et al. 2007; Neumeyer-Gromen et al. 2009). In nuclear industry workers, there was no association between lung cancer mortality and cumulative radiation exposure (lag 10) without adjustment for duration (Cardis et al. 2007). In diesel-exposed potash miners, adjusting for time since hire resulted in a stronger association between lung cancer mortality and exposure (Neumeyer-Gromen et al. 2009). In a study of autoworkers, Chevrier et al. (2012) assessed different analytical methods and also showed evidence of a healthy worker survivor effect for lung cancer.

Average exposure was not significantly associated with lung cancer risk. Estimated trucking industry EC exposures declined considerably over the course of the study and also varied based on location, job, and the period of diesel forklift use. Using model-based data to estimate trucking industry exposure trends (Davis et al. 2011), the median EC exposure over a shift for LH drivers in 1971–1980, 1981–1990, and 1991–2000 was 5.88 µg/m^3^, 4.26 µg/m^3^, and 2.01 µg/m^3^, respectively. For P&D drivers, in warm weather (windows open), estimated median levels were 9.59 µg/m^3^, 6.97 µg/m^3^, and 2.77 µg/m^3^, respectively, and in cold weather (windows closed), corresponding median levels were 4.15 µg/m^3^, 2.95 µg/m^3^, and 1.64 µg/m^3^, respectively. In the 1980s, the estimated median EC exposure for dockworkers working with diesel forklifts was 29.86 µg/m^3^, whereas for propane forklifts it was 1.43 µg/m^3^. Therefore, average exposure intensity is unlikely to be an accurate surrogate for cumulative exposure and pulmonary dose of particulate over time in the study cohort.

Exposure to vehicle exhaust particulate was estimated based on the assessment of EC mass in PM_1.0_. We conducted a source apportionment study using particle-phase organic molecular markers in personal and work area samples at a freight terminal in an urban area in 2003 (Sheesley et al. 2008a). These data indicated that for the LH drivers, P&D drivers, dockworkers, mechanics, and in the terminal yard and in an urban background site, most (≥ 80%) of the EC was from diesel exhaust sources, with a smaller percentage from spark ignition vehicles. These findings are consistent with other source apportionment studies indicating that diesel vehicles significantly contributed to EC in the United States during the period of the study, particularly in urban areas (Schauer 2003).

A unique feature of this study was the quantitative approach linking historical estimates of EC with job title and trucking terminal location information from work history records. We conducted a national exposure assessment at representative work locations in the participating companies. Statistical exposure models were developed that identified determinants of EC exposure, and these factors were used to estimate exposure nationally and historically (Davis et al. 2011). In contrast, previous studies of this industry estimated exposures based on diesel vehicle miles, emission rates, and fuel efficiency factors (Bailey et al. 2003; Steenland et al. 1998).

We conducted analyses with and without mechanics because we had less confidence in the historical extrapolation of their EC exposures since their job duties changed over time as major truck repairs were outsourced. Additionally, the patterns and potentially the composition of exposures for this job group were different from the others (McDonald et al. 2011). Misclassification of exposure would make it more difficult to detect a relationship between EC and lung cancer mortality with mechanics included. In addition, we hypothesized that because of their intermittent exposure, mechanics have less exposure to fresh particles that may be more hazardous than aged particles, thereby reducing their lung cancer risk. Short-lived reactive oxygen species (ROS) present in traffic-related PM and ultrafine particles resulting in DNA damage is a mechanism whereby traffic-related exposures may increase lung cancer risk. ROS activity has been found to be associated with exposure to traffic-related PM and ultrafine particles, potentially explaining the greater risk in persons such as drivers and dockworkers who are in jobs with more continuous exposures to fresh exhaust (de Kok et al. 2006; Li et al. 2003).

Our results are consistent with a large body of literature that supports a relationship between diesel exhaust exposure and lung cancer risk in occupationally exposed workers (Diesel Working Group 1995; Office of Research and Development 2002). These studies have not included historical quantitative estimates of exposure. The positive association between cumulative EC exposure and lung cancer estimated in the present analysis is consistent with our previous assessments of lung cancer mortality by job title in this cohort (Garshick et al. 2008) and is similar to results from a case–control study conducted by Steenland et al. (1990) in the same industry in 1982–1983. Further, our findings are consistent with an analysis that demonstrated a linear relationship between PM mass and lung cancer mortality based on estimates of PM mass exposure from particulate air pollution, second hand smoke, and active smoking (Pope et al. 2011).

Several other studies have measured diesel exhaust exposures and estimated lung cancer mortality in miners. A study of potash miners exposed to diesel exhaust with cumulative exposure based on occupational measurements was suggestive of a positive exposure–response relationship (Neumeyer-Gromen et al. 2009). Attfield et al. (2012) reconstructed historical EC exposures over a 30–50 year period for a cohort mortality study of 12,315 U.S. nonmetal miners. In comparison to the trucking industry, the duration of underground mine exposure was relatively short (a mean of 8 years compared with a mean of 21.6 years in the present study) but more intense, with mean EC exposures of 128 µg/m^3^ compared with the historical trucking industry’s lower cross-shift estimates noted previously. Cumulative exposure quartiles reported by Attfield et al. (2012) for underground miners ranged from < 108 µg/m^3^-years (equivalent to 1,296 µg/m^3^-months) to ≥ 946 µg/m^3^-years (equivalent to 11,352 µg/m^3^-months) in a 15-year exposure lag model, which overlaps with our estimates for trucking industry cumulative EC. For example, for the 10-year lag exposures excluding the mechanics, the 75th percentile was 1,305 µg/m^3^-months and the maximum was 10,341 µg/m^3^-months (Table 3). We converted the 10-year lag regression coefficient (indicating risk per 1,000 µg/m^3^-months) for trucking industry EC exposure in Table 5 to the same units used in Attfield et al. (2012) by multiplying by 12 months. This calculation resulted in an HR of 2.77 (95% CI: 0.85, 9.00) per 1,000 µg/m^3^-years. Attfield et al. (2012) found that the corresponding HR, based on a 15-year lag, was 4.06 per 1,000 µg/m^3^-years (95% CI: 2.11, 7.83), indicating overlap between the relative risks estimated for the two study cohorts.

As mentioned above, there is no one exposure metric unique to diesel exhaust; therefore, we selected EC mass in PM_1.0_ as our marker. Although our source apportionment analyses support this choice, using EC also incorporates exposure to other mobile sources (Sheesley et al. 2008b). In addition, it is also possible that other parameters related to vehicle exhaust particulate matter are relevant to the assessment of health risk, such as particle number or surface area (Wittmaack 2007). As the biologic mechanisms are not known, we do not propose that EC serves as a lung carcinogen, but serves as a marker of exposure to components associated with combustion sources.

An additional limitation is the lack of exposure information before employment in the four participating companies. In a survey mailed in 2003 to a sample of active and retired workers (Jain et al. 2006), workers may have had up to 10 additional years of trucking industry exposure, thereby underestimating actual exposure and potentially reducing apparent risk associated with estimated exposure. Potential overestimation of exposures using our exposure model for workers at very small terminals with little trucking activity would have a similar effect. Additionally, our cumulative exposure metric has the implicit assumption that effects of long-term low-intensity exposures are equivalent to effects of shorter-term high-intensity exposure resulting in a comparable estimate of cumulative exposure in terms of µg/m^3^-month. The overlap in estimated lung cancer risks in the current trucking industry cohort and the underground miner cohort study reported by Attfield et al. (2012) supports this assertion.

Although we obtained detailed work records and carefully assessed exposure, an additional limitation is a lack of information on personal risk factors for lung cancer. Although cigarette smoking is a major risk factor, the degree that it is a confounder depends on its differential association with exposure (Blair et al. 2007). Since workers in the study cohort were all unionized trucking industry employees, they were generally similar with regards to socioeconomic status which is a known correlate of smoking habits. Smoking rates also vary by age and birth cohort and all analyses were adjusted for these factors. In a 2003 questionnaire survey, we obtained information regarding smoking habits. In our previous assessment of lung cancer risk in this cohort based on job title (Garshick et al. 2008), indirect adjustment (Axelson and Steenland 1988) for smoking did not meaningfully influence estimated relative risks for lung cancer. Adjustment for differences in smoking habits based on job title increased relative risk estimates among P&D drivers, dockworkers, and combination workers by 4–8% and decreased relative risks among LH drivers by 15%. In addition, if short-term workers have different behaviors (e.g., smoke more heavily) than longer-term workers, this could contribute to negative confounding in analyses unadjusted for duration. Drivers have a commercial license and undergo medical certification that could also indicate that healthier workers continue working.

To conclude, our results suggest lung cancer mortality increases with cumulative EC exposures in trucking industry workers in jobs with regular exposures to particulates from pre-2007 diesel exhaust and other mobile sources. Our exposure assessment indicates that there has been substantial success in reducing trucking industry EC exposures and therefore we predict a reduction in future lung cancer risk in this industry.
